# Effective kissing stent to severe stenosis of the superior mesenteric artery replacing the common hepatic artery

**DOI:** 10.1186/s42155-018-0025-1

**Published:** 2018-09-25

**Authors:** Yusuke Date, Hiromasa Katoh, Takatoshi Abe, Hirhoshi Nagamine, Hiroiku Hara, Yushi Kawase

**Affiliations:** Department of Cardiovascular Surgery and Department of Cardiology, Yokohama Sakae Kyosai Hospital, 132, Katsuracho, Sakae-ku Yokohama-shi, Kanagawa 247-8581 Japan

**Keywords:** Chronic mesenteric ischemia, Pneumatosis intestinalis, Pneumoperitoneum, Kissing stent technique

## Abstract

**Background:**

Endovascular therapy (ET) for chronic mesenteric ischemia (CMI) is a effective treatment to relieve the symptoms, such as postprandial abdominal pain, food fear, and progressive weight loss. CMI is not known to be caused by rare anatomical variation of severe stenosis of the superior mesenteric artery (SMA), with replaced the common hepatic artery to the SMA. The treatment of such a rare anatomical variation using ET technique has not been discribed. ET with kissing stent technique can be applied to the CMI accompanied with a rare anatomical variation.

**Case presentation:**

An 80-year-old woman presented with a history of intermittent, severe epigastric pain. Over the preceding 5 months, she had less severe and self-resolving epigastric pain 15–30 min after every meal. Abdominal computed tomography (CT) showed severe calcification of the SMA origin and bubble-like intramural gas of the small bowel with the contrasted wall pneumoperitoneum. As the patient did not have peritonitis, a conservative approach was used. Angiography performed after symptom resolution showed severe stenosis of the SMA origin with calcification, and the SMA had replaced the common hepatic artery. ET with the kissing stent technique, namely stenting to the SMA and common hepatic artery, was successfully performed and relieved the patient’s symptoms.

**Conclusions:**

CMI cause the symptoms of Pneumatosis intestinalis (PI) and pneumoperitoneum. Severe stenosis of the SMA origin replacing the common hepatic artery is a rare anatomic variation, which can cause CMI symptoms. ET with a kissing stent is the effective treatment option for the mesenteric artery stenosis accompanied with such rare anatomical variation.

## Background

Endvascular therapy (ET) is the first treatment option for the symptomatic chronic mesenteric ischemia (CMI) to relieve the symtoms (Pecoraro et al. 2013; Oderich et al. 2009). Symptomatic patients have stenosis or occlusion of at least two of three mesenteric arteries because of rich collateral circulation (Gustavo and Oderich 2014). Symptoms of CMI are rarely caused by only one artery, the severe stenosis of SMA with replaced the common hepatic artery to the superior mesenteric artery (SMA). It has not yet been described of how to treat such this rare anatomical variation using the ET with the kissing stent technique. We report a patient with PI and pneumoperitoneum concomitant with CMI due to severe stenosis of the origin of the SMA, which replaced the common hepatic artery and effective ET with the kissing stent technique to treat this anatomical variation.

## Case presentation

**Fig. 1:**
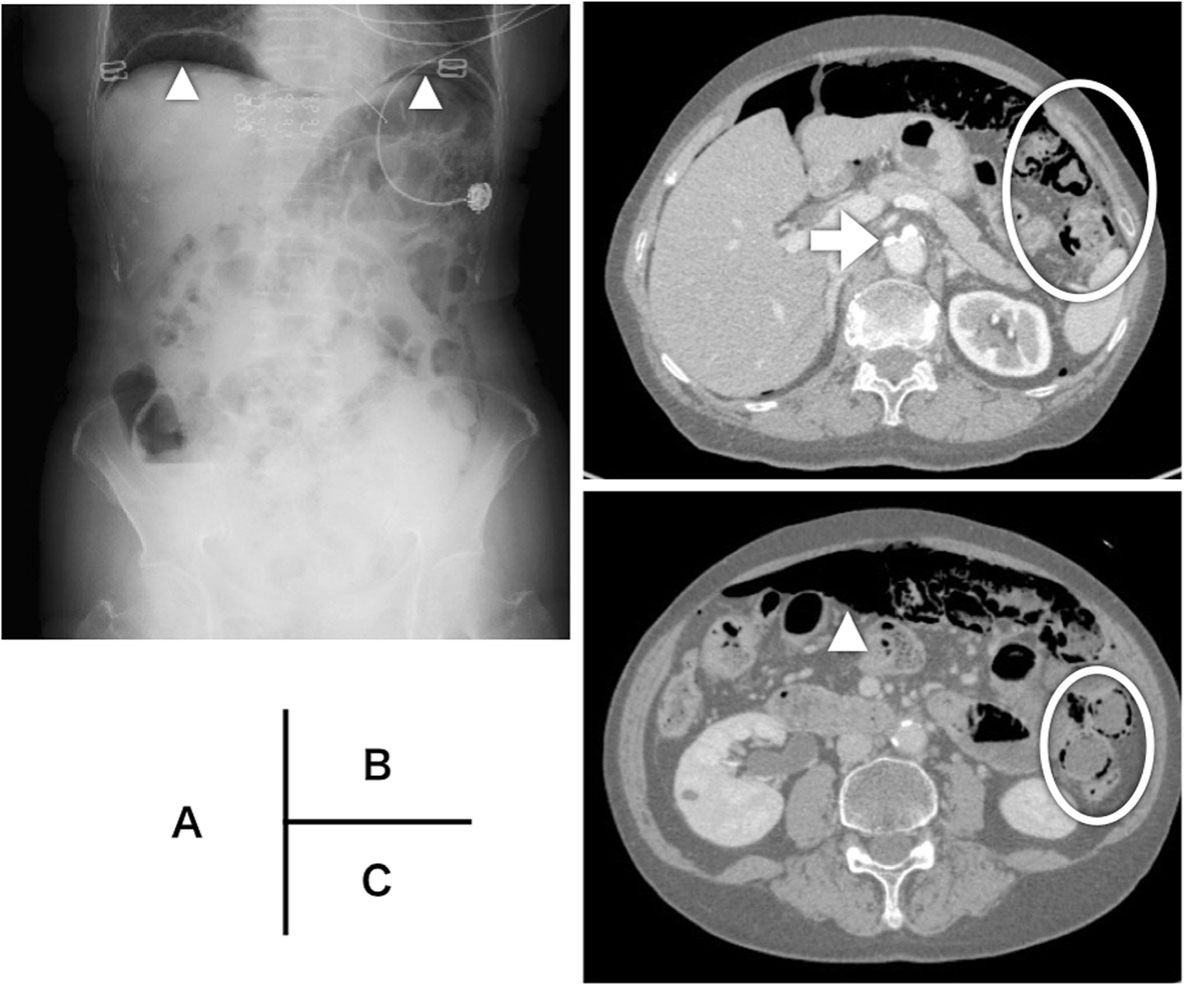
Abdominal plain radiography showing free air (**a**, triangle). Enhanced abdominal computed tomography showing severe calcification of the origin of the SMA (arrow), bubble-like intramural gas of the small bowel (circle), and pneumoperitoneum (triangle) (**b**, **c**)

An 80-year-old woman presented with a history of intermittent, severe epigastric pain. Over the preceding 5 months, she had less severe and self-resolving epigastric pain 15–30 min after every meal. The full blood count, serum biochemistry values, and C-reactive protein level were within normal ranges. Additionally, arterial gas analysis was normal. Abdominal plain radiography showed free air between the liver and diaphragm, and PI (Fig. 1a). Abdominal computed tomography showed severe calcification of the SMA origin; SMA peripheral flow was reserved (Fig. 1b). Computed tomography (CT) revealed bubble-like intramural gas of the small bowel with the contrasted wall pneumoperitoneum (Fig. 1c).

Since the patient did not have peritonitis, a conservative approach was performed. She was managed in the condition of intensive care unit, due to fears of the potential for acute mesenteric ischemia due to mesenteric artery occlusion or non-occlusive mesenteric ischemia. She was managed for bowel obstruction, which included fasting and intravenous fluid administration. She received heparin infusion to prevent SMA occlusion and maintain collateral flow. During admission, she reported abdominal pain relief. Seven days after admission, abdominal plain radiography showed improvement in PI and pneumoperitoneum; therefore, she was permitted to begin drinking fluids. There was no evidence of recurrent abdominal pain after the fluid consumption, so she was allowed to eat solid food.

**Fig. 2:**
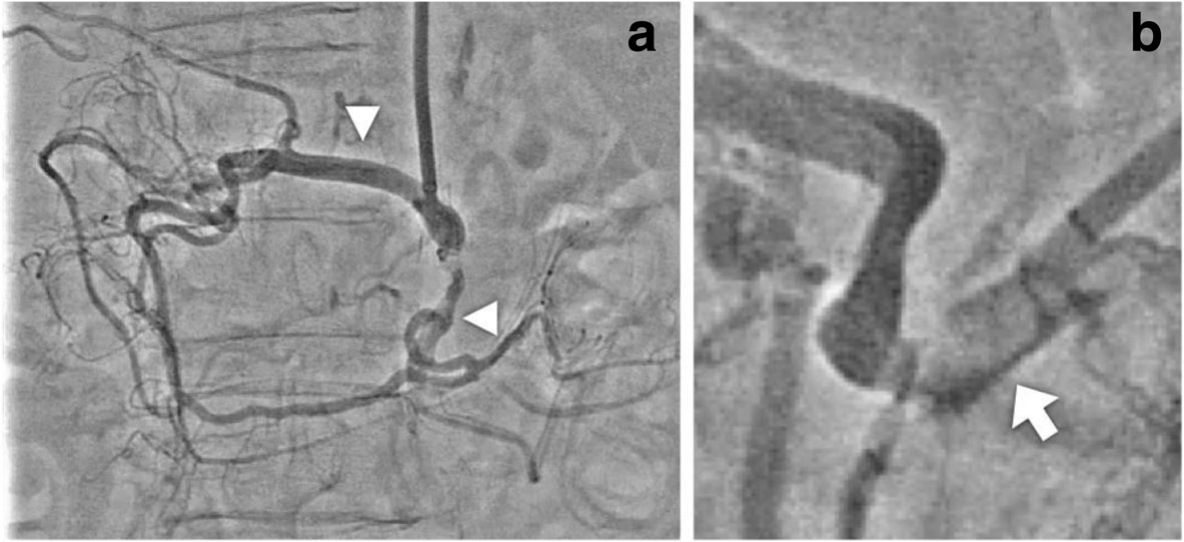
Angiography revealed the anatomical variations of the SMA replacing the common hepatic artery (**a**), and severe stenosis of the origin of the SMA with calcification (arrow) (**b**)

**Fig. 3:**
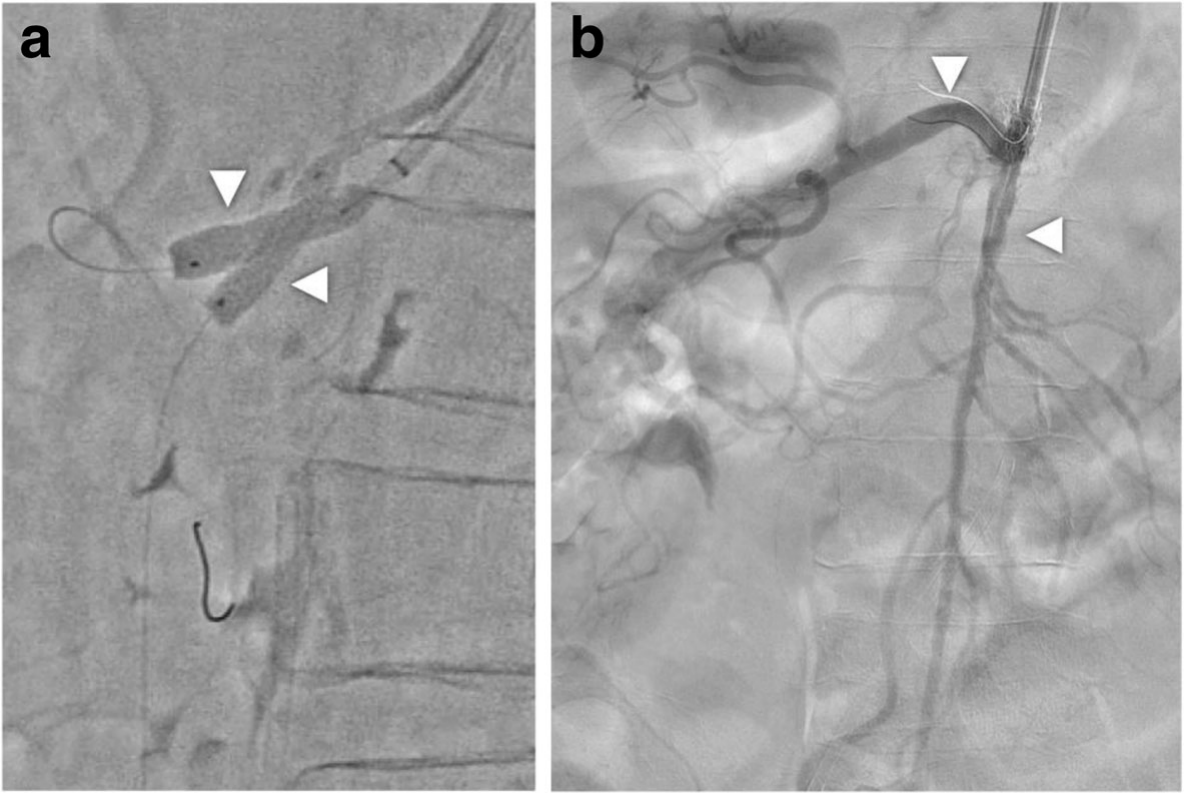
Stenting to the SMA and the common hepatic artery with the kissing stent technique was performed (**a**). The final angiography showed satisfactory circulation to the SMA and the common hepatic artery (**b**)

Angiography was planned to relieve the postprandial abdominal pain. The findings showed severe stenosis of the SMA origin with calcification, and the SMA had replaced the common hepatic artery (Fig. 2a, b). ET, namely stenting to the SMA and common hepatic artery, was performed. The SMA trunk was engaged with a 6F Parent Plus 60 guiding catheter (Medikit, Tokyo, Japan) from the left brachial artery. Initially, 8000 units of heparin was infused, and additional heparin was added to keep the activated whole blood clotting time over 200 s. The SMA occlusion was traversed using a 0.014” NEO EVT Guide Wire Cruise (ASAHI INTECC J-sales, Tokyo, Japan). The SMA trunk to the hepatic artery was traversed using a 0.014” NEO EVT Guide Wire Cruise, which was engaged with a 6F SheathLess PV (Cardian Health, Ohio, USA) from the right brachial artery. Intravascular ultrasonography (IVUS) (Navifocus WR, TERUMO, Tokyo, Japan) revealed severe stenosis of the SMA trunk with calcification. Balloon dilation was performed with the kissing ballon technique using a 4-mm Coyote ES (Boston Scientific, Marlborough, MA, USA) to the SMA and 5-mm SHIDEN RX (KANEKA MEDICAL, Osaka, Japan) to the common hepatic artery. After dilation, two balloon-expandable stents (5-mm PALMAZ Genesis (Cardian Health, Ohio, USA) to the SMA and 6-mm PALMAZ Genesis to the common hepatic artery) were implanted with the kissing stent technique from the common ostium (Fig. 3a). The sizes of the stents were based on the IVUS measurements. IVUS showed good orifice expansion. Final angiography showed satisfactory circulation to the terminal organ from the orifice (Fig. 3b).

Endovascular therapy relieved the patient’s symptoms. She required dual anti-platelet therapy (DAPT) with aspirin and clopidogrel post-intervention. Follow-up computed tomography showed patency of the orifice of the common hepatic artery and SMA, and there was no evidence of PI or pneumoperitoneum. No recurrent postprandial abdominal pain occurred for two years after the ET.

## Discussion

The kissing stent technique is very useful in cases of the severe SMA stenosis with a rare anatomical variation wherein the SMA replaced common hepatic artery. Such anatomical variation causes the symptoms of CMI, although only one of three main mesenteric artery was involved.

The classic symptoms of CMI include abdominal pain, weight loss, and food fear (Gustavo and Oderich 2014). CMI rarely occurs concomitantly with PI and pneumoperitoneum, and the radiographic signs often indicate acute mesenteric ischemia (Yukaya et al. 2014). There are only a few cases of CMI presentation. Nicholas Dawe et al. reported a CMI case with PI and pneuemoperitoneum (Wayne et al. 2010). In our case, the symptoms of PI and pneumoperitoneum were caused by CMI, because there was no evidence of mesenteric artery occlusion nor intestinal ischemia.

As PI and pneumoperitoneum are often life-threatening if untreated, urgent surgical intervention is required (Dawe and Akhtar 2010; Greenstein et al. 2007). In our case, there were no signs of occluded mesenteric arteries or abdominal peritonitis; thus, non-surgical therapy was selected. Our patient received repeated and careful observation to evaluate the conservative management.

The mesenteric circulation is rich in collateral networks between the three main visceral artery territories and internal iliac arteries. Most symptomatic patients with CMI have significant stenosis or occlusion of at least two of the three mesenteric arteries (Gustavo and Oderich 2014). Michels reported that the percentage of the common hepatic arteries arising from the SMA is 2.5% (Michkels 1966).

Our patient had mesenteric ischemia due to severe stenosis of the origin of the common hepatic artery and SMA. If the common hepatic artery had not replaced the SMA, PI and pneumoperitoneum could not have occurred, because there is rich collateral flow between the celiac artery and SMA. This rare variation of the common hepatic artery and the SMA caused CMI symptoms due to severe stenosis of the two mesenteric arteries.

PI is a sign of underlying disease (St. Peter et al. 2003). One of many non-surgical causes of pneumoperitoneum is PI (Wayne et al. 2010). The pathogenesis is hypothesized to be a relationship of the multifaceted factors causing PI (St. Peter et al. 2003). One of these factors is vascular disease as an underlying disease. Vascular disease causes mucosal injury and increasing bacteria gas production due to a defect in the mucosal immune barrier. This pathologic combination induces gas translocation to the intramural compartment and peritoneum.

In our case, severe stenosis of the SMA origin replacing the common hepatic artery caused prolonged, reduced blood flow to the intestine. This contributed to mucosal injury. Over the past 5 months when CMI symptoms occurred, delayed stenosis treatment caused PI and pneumoperitoneum. If the SMA origin was occluded, necrosis of the intestine, a sign of acute mesenteric ischemia, would develop.

Revascularrization is indicated in patients with CMI symptoms to relieve symptoms and prevent bowel infarction (Pecoraro et al. 2013). ET and open bypass strategy is performed in patients with CMI. Angioplasty with stenting is superior to open bypass as the first treatment option (Oderich et al. 2009). In our case, ET was selected, because it is a minimally invasive strategy. In addition, to preserving blood flow to the SMA and the common hepatic artery, the kissing stent technique was performed to avoid potential risks, such as occlusion or embolization of another vessel. The kissing stent technique is common for complex aortoiliac bifurcation treatment. Many reports have demonstrated the efficacy of the technique (Houston et al. 2007). However, to our knowledge, there are no reports of the kissing stent technique used in the SMA and the common hepatic artery.

It is uncertain whether patients will need DAPT to prevent stent thrombosis. However, in our case, DAPT was continued for 12 weeks, after which aspirin was continued. Performing ET is more likely to develop restenosis and to undergo another intervention (Oderich et al. 2009). It is important to observe patients carefully because of this rare adverse effect when using ET with the kissing stent technique.

## Conclusion

Severe stenosis of the SMA origin replacing the common hepatic artery is a rare anatomic variation, which can cause CMI symptoms. We also recommend ET with the kissing stent technique as the first treatment option for mesenteric artery stenosis with this anatomical variation.

## Abbreviations

CMI: Chronic mesenteric ischemia
CT: Computed tomography
DAPT: Dual anti-platelet therapy
ET: endovascular therapy
IVUS: Intravascular ultrasonography
PI: Pneumatosis intestinalis
SMA: Superior mesenteric artery
